# Saengmaeksan, a traditional polyherbal formulation containing *Panax ginseng*, improves energy metabolism during exercise

**DOI:** 10.1371/journal.pone.0296487

**Published:** 2024-01-29

**Authors:** Suji Baek, Jisu Kim, Myung Hee Nam, Sun Mi Park, Tae Sup Lee, Seo Young Kang, Ji-Young Kim, Hai-Jeon Yoon, Seung Hae Kwon, Jonghoon Park, Sang Ju Lee, Seung Jun Oh, Kiwon Lim, Bom Sahn Kim, Kang Pa Lee, Byung Seok Moon

**Affiliations:** 1 Research & Development Center, UMUST R&D Corporation, Seoul, Korea; 2 Physical Activity and Performance Institute (PAPI), Konkuk University, Seoul, Korea; 3 Department of Sports Medicine and Science in Graduated School, Konkuk University, Seoul, Korea; 4 Seoul Center, Korea Basic Science Institute, Seoul, Korea; 5 Department of Nuclear Medicine, Ewha Womans University College of Medicine, Seoul, Korea; 6 Division of RI Applications, Korea Institute Radiological and Medical Sciences, Seoul, Korea; 7 Department of Physical Education, Korea University, Seoul, Korea; 8 Department of Nuclear Medicine, Asan Medical Center, University of Ulsan College of Medicine, Seoul, Korea; Northwest University, UNITED STATES

## Abstract

Saengmaeksan (SMS), a representative oriental medicine that contains *Panax ginseng* Meyer, *Liriope muscari*, and *Schisandra chinensis* (1:2:1), is used to improve body vitality and enhance physical activity. However, there is limited scientific evidence to validate the benefits of SMS. Here, we investigated the in vitro and in vivo regulatory effects of SMS and its constituents on energy metabolism and the underlying molecular mechanisms. For this, quantitative real-time polymerase chain reaction, 3D holotomographic microscopy, western blotting, and glucose uptake experiments using ^18^F-fluoro-2-deoxy-D-glucose (^18^F-FDG) were performed using L6 cells to investigate in vitro energy metabolism changes. In addition, ^18^F-fluorocholine (^18^F-FCH) and ^18^F-FDG positron emission tomography/computed tomography (PET/CT) analyses, immunohistochemistry, and respiratory gas analysis were performed in mice post-endurance exercise on a treadmill. In the energy metabolism of L6 cells, a significant reversal in glucose uptake was observed in the SMS-treated group, as opposed to an increase in uptake over time compared to the untreated control group. Furthermore, *P*. *ginseng* alone and SMS significantly decreased the volume of lipid droplets. SMS also regulated the phosphorylation of extracellular signal-regulated kinase (ERK), phosphorylation of p38, mitochondrial morphology, and the expression of apurinic/apyrimidinic endonuclease 1/redox factor-1 (APE/Ref-1) in H_2_O_2_-stimulated L6 cells. In addition, SMS treatment was found to regulate whole body and muscle energy metabolism in rats subjected to high-intensity exercise, as well as glucose and lipid metabolism in skeletal muscle. Therefore, SMS containing *P*. *ginseng* ameliorated imbalanced energy metabolism through oxidative stress-induced APE/Ref-1 expression. SMS may be a promising supplemental option for metabolic performance.

## Introduction

Imbalanced energy metabolism increases the risk of representative metabolic disorders, such as type 2 diabetes, obesity, and non-alcoholic fatty liver disease [1]. Oxidative stress and changes in energy metabolism induced by reactive oxygen species (ROS) play pathophysiological roles in metabolic syndrome [2]. Under oxidative stress conditions, ROS play an important role in cellular responses to oxidative stress-induced protein kinases, such as extracellular signal-regulated kinase (ERK) 1/2, p38, and c-Jun N-terminal kinase (JNK) [3, 4]. Therefore, inhibition of ROS- and oxidative stress- induced transcriptional signaling cascades is a potential strategy for homeostasis regulation.

The multifunctional protein apurinic/apyrimidinic endonuclease 1/redox factor-1 (APE1/Ref-1) plays a major role in energy metabolism and regulation of body fat and carbohydrate metabolism via activation of the antioxidant system [5]. In the presence of ROS-induced cellular signals generated during adenosine triphosphate (ATP) production, APE1/Ref-1 regulates transcription and potently suppresses ROS production. APE1/Ref-1 also plays an important role in ROS-induced mitochondrial fission or AP-1 site in the nucleus [6, 7].

*Panax ginseng* Meyer, the plant of Korean ginseng, has been used as a core herbal medicine for thousands of years and is currently a representative Korean natural product that is used worldwide as a functional food and medicine [8]. Numerous studies on energy metabolism regulation and improvement in exercise capacity using natural products have been conducted in recent times. Since energy metabolism disorders are multi-targeted diseases, the use of natural substances containing physiologically diverse active constituents is essential for treatment. *P*. *ginseng* has long been used as a single decoction prescription medicine and in polyherbal formulations for its synergistic effects [9]. *Schisandra chinensis* (SC) has been found to promote energy metabolism [10]. Intake of SC for six weeks improved endurance exercise capacity, increased the expression of Carnitine palmitoyltransferase 1b (CPT1b), which plays a vital role in fat metabolism, and expression of peroxisome proliferator-activated receptor gamma co-activator-1α (PGC-1α) involved in mitochondrial biogenesis. In addition, *Liriope muscari* (LM) was also confirmed to be associated with energy metabolism. Previous studies have demonstrated that LM intake reduced ROS levels [11], and the combination of LM and high-fat intake for 8 weeks significantly decreased serum total cholesterol (TC), triglyceride (TG), and low-density lipoprotein cholesterol (LDL-C) levels, and downregulated the expressions of peroxisome proliferator-activated receptor γ (PPARγ) and fatty acid synthase (FAS) in the adipose and liver of the mice [12].

Saengmaeksan (SMS), a prescription medicine containing *P*. *ginseng* (GS), *Schisandra chinensis* (SC), and *Liriope muscari* (LM), improves physical stamina [13]. SMS is prescribed to increase body vitality and enhance physical performance; however, scientific evidence to validate the efficacy of SMS has not been established. Therefore, in this study, we investigated and compared the antioxidant effects of GS alone and polyherbal SMS. We also evaluated the in vitro and in vivo potential of SMS to enhance athletic performance and inhibit free-radical generation during exercise.

## Materials and methods

### Materials

Fetal bovine serum (FBS), Dulbecco’s Modified Eagle Medium (DMEM), and penicillin-streptomycin (P/S) were purchased from Welgene (Gyeongsangbuk-do, Korea). MitoTracker^™^ Red FM dye was purchased from Thermo Fisher Scientific (Waltham, MA, USA). Antibodies against phosphorylated ERK 1/2 (P-ERK 1/2), ERK 1/2, phosphorylated p38 (P-p38), p38, and β-actin were purchased from Cell Signaling Technology (Danvers, MA, USA). Manufacturing grade-certified (Korean Pharmacopoeia) SMS was obtained from Jungwoo Medicine (Chungcheonnam-do, Korea). SMS is composed of 1,250 mg GS, 1,250 mg SC, and 2,500 mg LM in 100 mL water. All other reagents were purchased from Sigma-Aldrich (St. Louis, MO, USA).

### Cell culture and viability

L6 cells were obtained from the Korean Cell Line Bank (Seoul, Korea) and cultured according to the method described in an earlier study [14]. L6 cells were grown in DMEM containing 10% FBS and 1% P/S at 37 ± 2°C and in a 5% CO_2_ atmosphere. The cells were seeded in a 96-well plate (1 × 10^4^ cells/well) and incubated in the presence or absence of hydrogen peroxide (H_2_O_2,_ 300 μM) and 0.125 mg/mL GS or 0.5 mg/mL SMS for 24 h. To evaluate cell viability, the cells were subsequently incubated with 2-(4-iodophenyl)-3-(4-nitrophenyl)-5-(2,4-disulfophenyl)-2H-tetrazolium (WST-1) reagent for 2 h. The absorbance was recorded at 450 nm using a microplate reader (Bio-Rad, Hercules, CA, USA).

### In vitro glucose uptake analysis using ^18^F-fluoro-2-deoxy-D-glucose (^18^F-FDG)

L6 cells (1 × 10^5^ cells/well) were seeded in 6-well culture plates and treated with 0.125 mg/mL GS, 0.25 mg/mL LM, 0.125 mg/mL SC, GS + LM, GS + SC, LM + SC, or 0.5 mg SMS (mixture of GS, LM, and SC in the ratio of 1:2:1) in serum-free media. After 24 h, the cells were washed two times using phosphate buffered saline (PBS) and then treated with ~111 kilobecquerel (kBq) of ^18^F-FDG/mL serum free medium and incubated for 10, 30, 60, and 120 min at 37°C in a 5% CO_2_ atmosphere. At each time point, the medium was removed and the cells were washed using PBS. The adherent cells were harvested using 0.5% sodium dodecyl sulfate (SDS) and their radioactivity was determined using a 2470 Wizard^2^ gamma counter (PerkinElmer, MA, USA).

### Lipid volume analysis using 3D holotomography

L6 cells (1 × 10^5^ cells/mL) were seeded into a TomoDish, incubated for 24 h, and then treated with 0.125 mg/mL GS or 0.5 mg/mL SMS for 24 h. The lipid droplet and cell volumes were observed and measured using a holotomography microscope (HT-1H; Tomocube, Daejeon, Korea) and quantified using TomoStudio^™^ software. The volume ratio represents the ratio of lipid volume to cell volume.

### Periodic acid-Schiff (PAS) staining

L6 cells (1 × 10^5^ cells/mL) were seeded into a 12-well plate, incubated for 24 h, and then treated with 0.125 mg/mL GS or 0.5 mg/mL SMS for 24 h. The cells were fixed with 4% formalin for 15 min, followed by staining with 0.5% periodic acid solution for 10 min. Next, the cells were incubated with Schiff’s reagent for 10 min at room temperature and stained with hematoxylin for 2 min. The cells were observed under a microscope.

### Quantitative real-time polymerase chain reaction (qPCR)

Total RNA was extracted from L6 cells and mice soleus muscles using TRIzol reagent [15]. cDNA was synthesized using the Superscript III First Strand Complementary DNA synthesis kit (Thermo Fisher Scientific). An Applied Biosystems 7500 Fast Real-Time PCR System (Thermo Fisher Scientific) and SYBR Green PCR mix were used to perform qPCR. The primer sequences used were: Rattus CPT1: 5′-GTG CTG GAG GTG GCT TTG GT-3′ (forward) and 5′-TGCTTGACGGATGTGGTTCC-3′ (reverse); Rattus β-actin: 5′-GGC CAA CCG TGA AAA GAT G-3′ (forward) and 5′-GGA TCT TCA TGA GGT AGT CTG TC-3′ (reverse); Mus musculus GLUT4: 5′-AGA GTC TAA AGC GCC T-3′ (forward) and 5′-CCG AGA CCA ACG TGA A-3′ (reverse); M. musculus CD36: 5′-CGG CGA TGA GAA AGC AGA-3′ (forward) and 5′-ACT CCA ACA CCA AGT AAG ACC A-3′ (reverse); M. musculus PGC-1α: 5′-TCT GGA ACT GCA GGC CTA ACT C-3′ (forward) and 5′-TCT GGA ACT GCA GGC CTA ACT C-3′ (reverse); and M. musculus β-actin: 5′-GGC CAA CCG TGA AAA GAT G-3′ (forward) and 5′-GGA TCT TCA TGA GGT AGT CTG TC-3′ (reverse). The qPCR protocol involved an initial denaturation step at 95°C for 10 min, followed by 40 cycles of denaturation at 95°C for 10 s, annealing at 60°C for 30 s, and extension at 72°C for 30 s. Relative mRNA levels were calculated using the 2^-ΔΔCt^ method and were normalized using β-actin mRNA levels.

### Mitochondrial morphology analysis

L6 cells (1 × 10^4^ cells/mL) were seeded into a confocal dish and treated with H_2_O_2_ (300 μM), H_2_O_2_ and GS (0.125 mg/mL), or H_2_O_2_ and SMS (0.5 mg/mL) for 24 h. The cells were then stained using MitoTracker^™^ Red FM (200 nM) for 40 min. After washing the cells three times using PBS, they were observed under a fluorescence microscope (Laser scanning microscopes 780, Zeiss, Oberkochen, Germany). Mitochondrial length was measured for more than twenty cells according to an earlier study and analyzed using ImageJ software (Version 1.52a, United States National Institutes of Health, Bethesda, MD, USA) [15].

### Immunocytochemistry

Immunocytochemistry was performed as described in an earlier study [15]. L6 skeletal muscle cells (5 × 10^3^ cells/mL) were seeded in 8-well chambers and incubated in the absence or presence of serum for 24 h, followed by treatment with GS (0.125 mg/mL) or SMS (0.5 mg/mL) for 24 h. The cells were fixed and permeabilized in 4% formalin and 0.1% Triton X-100 for 10 min, and then sequentially incubated with anti-APE/Ref-1 antibody (1:1000) and Alexa Fluor 488-conjugated secondary antibody (excitation and emission wavelength, 492 nm and 527 nm, respectively) for 1 h. Images were captured using a K1-fluo microscope (Nanoscope system, Daejeon, Korea). The nuclei were stained using 4′,6-diamidino-2-phenylindole (excitation and emission wavelength, 358 nm and 461 nm, respectively). Fluorescence intensity was measured and analyzed using ImageJ software.

### Immunoblotting

Immunoblotting was performed according to the method described in our previous study [16]. L6 cells were seeded into a 100-mm dish and treated with H_2_O_2_ (300 μM), co-treated with H_2_O_2_ and GS (0.125 mg/mL), or co-treated with H_2_O_2_ and SMS (0.5 mg/mL) or co-treated with H_2_O_2_ and PD98059 (10 μM) or co-treated with H_2_O_2_ and SB203580 (10 μM) for 30 min. Post-cell lysis, proteins were separated using 12% polyacrylamide gel electrophoresis and transferred onto polyvinylidene fluoride (PVDF) membranes. The PVDF membranes were blocked using 5% bovine serum albumin (BSA) for 1 h at room temperature and incubated with specific antibodies against P-ERK 1/2, ERK 1/2, P-p38, p38, and β-actin for 16 h at 4°C. Membranes were then incubated with a secondary antibody (conjugated with horseradish peroxidase) for 1 h. Protein levels were determined using chemiluminescence and analyzed using ImageJ software.

### Animal care and energy metabolism tests

Animal care and experimentation were performed in accordance with the institutional guidelines. The Ethics Committee of the Konkuk University Institutional Animal Care and Use Committee (KU19149, 6 Sep 2019) approved the study protocol. The experimental animals (male 8-week-old ICR mice) were housed in standard cages in a breeding room maintained at a constant temperature of 25 ± 2°C, a humidity of 55 ± 5%, and a 12 h light/dark cycle, and provided with a standard feed (5L79 formula) containing 18% protein, 0.85% calcium, and 0.62% phosphorus (Orient Bio Inc., Gyeonggi-do, Korea). Energy metabolism during exercise was measured in a metabolic chamber. The mice were administered SMS (10 mL/kg) 30 min prior to exercise. The exercise conditions inside the metabolic chamber included 18 m/min, 8° slope, and 70% of maximum VO_2_ max [17]. The flow rate was kept constant at 3 L/min and energy metabolism was measured for 1 h. Respiratory gas was measured using an open-circuit apparatus in accordance with the method proposed in an earlier study [18].

### ^18^F-FDG and ^18^F-FCH PET/CT analyses

To investigate changes in ^18^F-FDG and ^18^F-FCH uptake in the skeletal muscles of exercised mice, PET/CT imaging was performed according to the method described in our earlier studies [14, 15]. The mice (male, 8-week-old, 33.9 ± 1.8 g) were fasted for 6 h prior to PET imaging and exercised on a treadmill according to the same protocol involving the respiratory gas measurements described above. Mice were randomly divided into three groups (n = 8 in each group): untreated group (UN, maintained under standard conditions); a group orally administered distilled water and exercised for 1 h (EX); and a group orally administered SMS (10 mL/kg) for 30 min before exercise and then exercised for 1 h (EX + SMS). Mice in all three groups were intravenously administered a single dose of ^18^F-FDG (8.65 ± 0.81 MBq) or ^18^F-FCH (9.68 ± 0.39 MBq), following which PET imaging was performed using a dedicated small animal PET/CT scanner (nanoScan PET/CT, Mediso Medical Imaging Systems, Budapest, Hungary). The mice were first anesthetized using 2.5% isoflurane in 7:3 N_2_/O_2_ and then images were acquired by scanning for 20 min after 60 min or 40 min of conscious ^18^F-FDG or ^18^F-FCH uptake, respectively. CT scans were used for attenuation correction and anatomical localization of PET signals. The acquired PET images were reconstructed using the 3D Adjoint Monte Carlo method with scatter and random corrections. Volumes-of-interest (VOIs) were drawn on the CT images of individual animals in a slice-by-slice manner to analyze the uptake in both mid-lower legs in the summed image. Regional uptake of radioactivity was decay-corrected to the injection time and expressed as the mean maximum standardized uptake value (SUV_max_), which was normalized to the amount of radioactivity injected and the animal’s body weight. InterView Fusion software (v3.03.089.0000, Mediso Medical Imaging Systems, Budapest, Hungary) was used to analyze the standardized uptake values in the VOIs after reconstruction and quantification.

### Statistical analysis

All statistical analyses were performed using Prism software (version 4.0; GraphPad Software, La Jolla, CA, USA). Quantitative data were expressed as the mean ± standard deviation (SD), and comparisons of quantitative data between two groups were analyzed using an unpaired t-test. Statistical significance was set to *P* <0.05.

## Results

### Effect of SMS composition on glucose/fatty acid metabolism in L6 skeletal muscles

**Fig 1:**
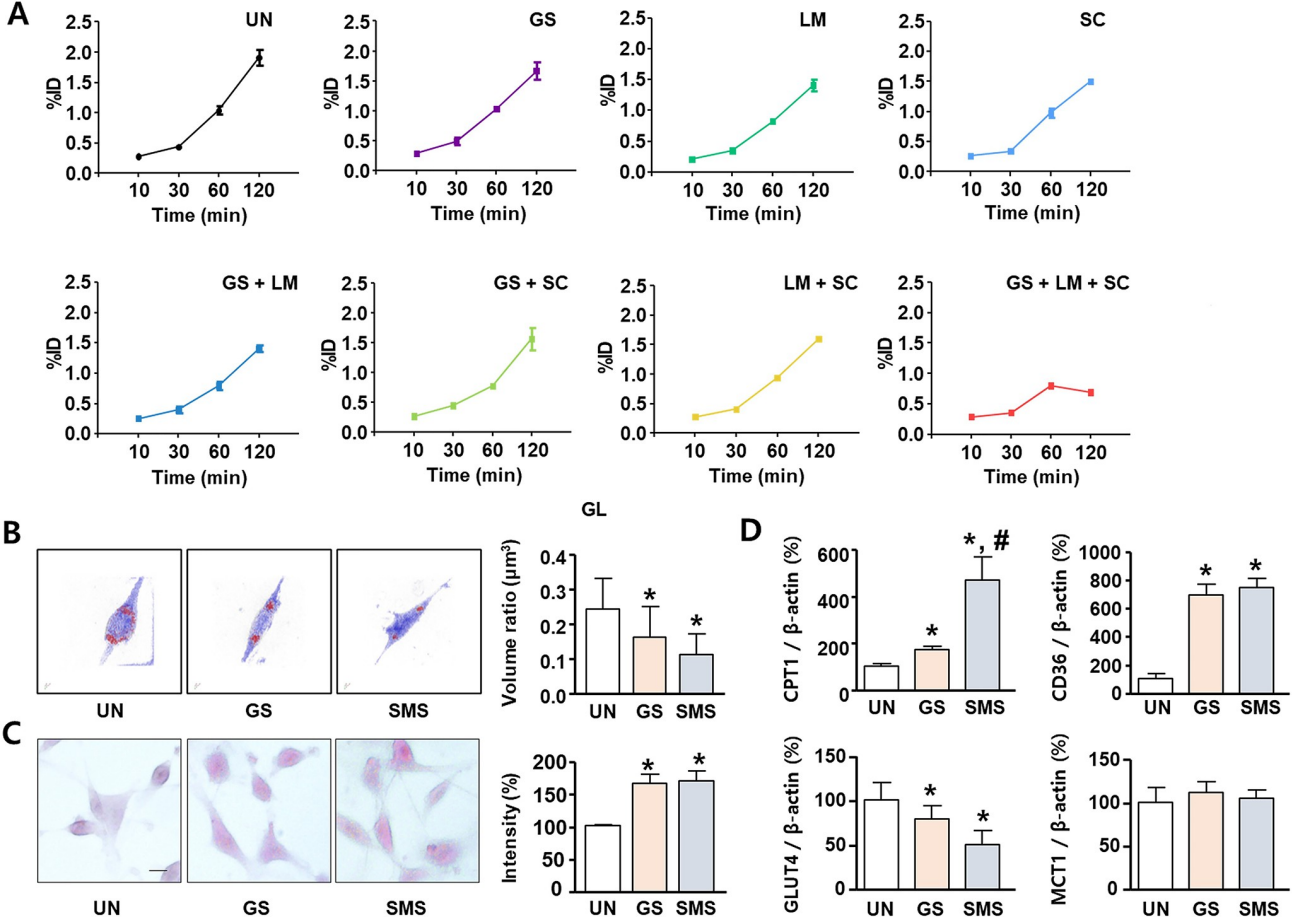
Effect of SMS on glucose and fat metabolism in L6 cells. (A) L6 cells were treated with 0.125 mg/mL GS, 0.25 mg/ mL LM, 0.125 mg/ mL SC, GS + LM, GS + SC, LM + SC, or SMS (mixture of GS, LM, and SC in the ratio of 1:2:1) for 24 h, and then incubated with ^18^F-fluoro-2-deoxy-D-glucose (^18^F-FDG) for 10, 30, 60, and 120 min. The graph represents ^18^F-FDG cell uptake determined using a gamma counter and the data are expressed as %ID. (B) Lipid droplet volume analyzed using 3D hologram microscopy. L6 cells were incubated in the presence or absence of GS (0.125 mg/mL) or SMS (0.5 mg/mL) in serum-free media for 24 h. (C) Glycogen store was measured using by PAS staining. (D) mRNA expression levels of CPT1, CD36, GLUT4 and MCT1 normalized to that of β-actin in L6 cells. All data are expressed as mean percentages relative to the UN group ± SD. *P < 0.05 compared to the UN group. ^#^*P* < 0.05 compared to the GS group. UN: untreated, H_2_O_2_: hydrogen peroxide treated, GS: ginseng, SMS: saengmaeksan.

SMS consists of GS, LM, and SC. Therefore, the animals used for the glucose uptake experiments were divided into single treatment and mixed treatment groups. Glucose uptake analysis was performed in L6 skeletal muscle cells using ^18^F-FDG to evaluate the effect of SMS composition on gluconeogenesis regulation. Radioactivity was determined using a gamma counter. L6 cells were treated with SMS in an FBS-deficient medium for 24 h. The percentage injected dose (%ID) indicated that metabolism markedly increased in the group with L6 cells (UN) (Fig 1A; S1 Table). In addition, the treatment groups, including animals treated with individual constituents or a combination of constituents, exhibited no significant differences in glucose uptake compared with that of the UN group. A pattern of significant reversal of uptake, which increased over time, was observed in the group treated with only SMS as shown in Fig 1A (at 120 min; 1.91 ± 0.12%ID in the UN group, 0.69 ± 0.01%ID in the SMS group, and 1.41 ± 0.06 to 1.67 ± 0.14%ID in the other groups). A similar pattern of significant uptake reversal was also observed in myoblast C2C12 cells (S1 File). The groups that showed significant differences in glucose uptake were analyzed for changes in fat metabolism in UN, and GS- or SMS-treated L6 cells. In lipid droplet analysis performed by 3D hologram microscopy, L6 cells treated with GS and SMS exhibited significantly reduced lipid droplet volume ratios of 0.16 ± 0.09 and 0.11 ± 0.06, respectively (Fig 1B; S1 Table). In glycogen store analysis performed by PAS staining, L6 cells treated with GS and SMS exhibited significantly increased the PAS-positive cells of 165.7 ± 15.4 and 169.4 ± 16.6, respectively (Fig 1C; S1 Table). To confirm GS and SMS whether can regulate the lipid and glucose metabolism, the mRNA expression of CPT1, CD36, GLUT4 and MCT1 were tested. GS regulated the expression of CPT1, CD36, GLUT4 and MCT1 by 171.7 ± 13.7%, 697.4 ± 74.7%, 79.4 ± 15.6% and 111.9 ± 12.0%, respectively. SMS regulated the expression of CPT1, CD36, GLUT4 and MCT1 by 471.2 ± 96.6%, 744.9 ± 64.5%, 50.5 ± 16.0% and 105.2 ± 9.1%, respectively (Fig 1D; S1 Table).

### Effects of GS and SMS on H_2_O_2_-induced oxidative stress in L6 cells

**Fig 2:**
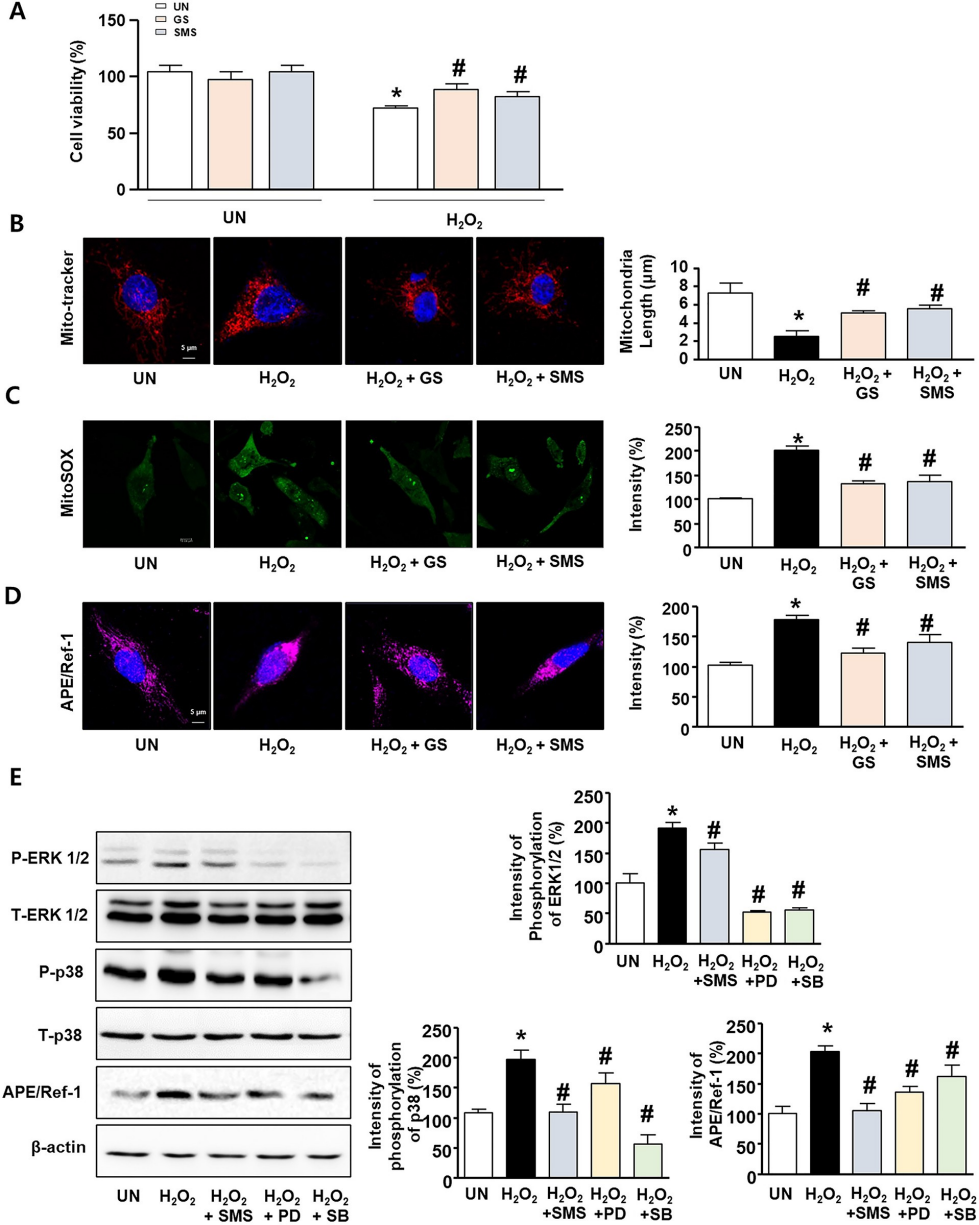
Antioxidant effects of GS and SMS on H_2_O_2_-stimulated L6 cells. (A) Cell viability (WST-1 reagent) of L6 cells (1 × 10^4^ cells/mL) treated with GS or SMS for 24 h in the presence or absence of H_2_O_2_ (300 μM). Data are expressed as mean percentages relative to the UN group. (B) L6 cells co-treated with GS or SMS for 24 h in the presence or absence of H_2_O_2_, and stained using MitoTracker^™^ Red FM. Mitochondrial morphology was observed using a fluorescence microscope and mitochondrial length was measured using ImageJ software. (C) L6 cells co-treated with GS or SMS in the presence or absence of H_2_O_2_ for 24 h, and then stained with MitoSOX. The graph indicates the intensity of MitoSOX. (D) Cells stained using anti-APE/Ref-1 (pink) and DAPI (blue). (E) Western blot analysis of L6 cells treated with SMS (0.5 mg/mL) or PD98059 (10 μM) or SB203580 (10 μM) for 30 min in the presence or absence of H_2_O_2_ (300 μM), and quantification of P-ERK 1/2, p38, and APE/Ref-1 expression relative to UN (100%). All data are expressed as mean ± SD. *P < 0.05 compared to the UN group. ^#^*P <* 0.05 compared to the group treated with only H_2_O_2_. UN: untreated, H_2_O_2_: hydrogen peroxide treated, GS: ginseng, SMS: saengmaeksan.

To determine the effect of GS and SMS on H_2_O_2_-induced oxidative stress in L6 cells, we performed a cell viability assay and evaluated mitochondrial morphology. L6 cells were treated with GS (300 μg/mL) or SMS (300 μg/mL) in the absence or presence of H_2_O_2_ (300 μM) for 24 h. First, considering the relative cell viability as 100% of the cell viability in the UN group, no cytotoxicity was observed in the groups treated with GS and SMS. In contrast, a significant reduction in cell viability to 72.4 ± 1.9% was observed in the H_2_O_2_-treated group (Fig 2A; S2 Table). However, the H_2_O_2_ + GS and H_2_O_2_ + SMS groups increased cell survival compared to H_2_O_2_. An immunocytochemistry assay revealed that H_2_O_2_ treatment significantly altered the fission form of mitochondrial morphology. However, mitochondrial morphology after treatment with H_2_O_2_ + GS or H_2_O_2_ + SMS was similar to that observed in the UN group. The mean mitochondrial length in the H_2_O_2_ group was 2.5 ± 0.6 μm, compared with 7.3 ± 1.0 μm, 5.4 ± 0.2 μm, and 5.5 ± 0.4 μm, in the UN, H_2_O_2_ + GS, and H_2_O_2_ + SMS groups, respectively (Fig 2B; S2 Table). H_2_O_2_ significantly induced ROS expression compared to the expression level in the quiescent state, whereas SMS significantly inhibited ROS expression (Fig 2C; S2 Table). In addition, we investigated APE/Ref-1 expression levels using an immunocytochemistry assay. GS and SMS significantly decreased H_2_O_2_-induced APE/Ref-1 overexpression (Fig 2D; S2 Table). L6 cells were treated with and without SMS or H_2_O_2_ and subjected to western blot analysis. H_2_O_2_ significantly induced P-ERK 1/2 (191.0 ± 10.1%) expression as compared with the expression level in the quiescent state. In contrast, SMS significantly inhibited H_2_O_2_-induced phosphorylation of ERK 1/2 and p38, similar to the inhibitors, PD98059 and SB203580. The expression levels of phosphorylated ERK 1/2 and p38 upon treatment with SMS were 155.4 ± 10.9% and 101.8 ± 12.4%, respectively. Furthermore, APE/Ref-1 expression in H_2_O_2_-stimulated L6 cells was significantly increased to 202.9 ± 9.7%. SMS significantly downregulated H_2_O_2_ -induced increased APE/Ref-1 expression (Fig 2E; S1 File; S2 Table).

### SMS regulates carbohydrate/fat metabolism during acute exercise

**Fig 3:**
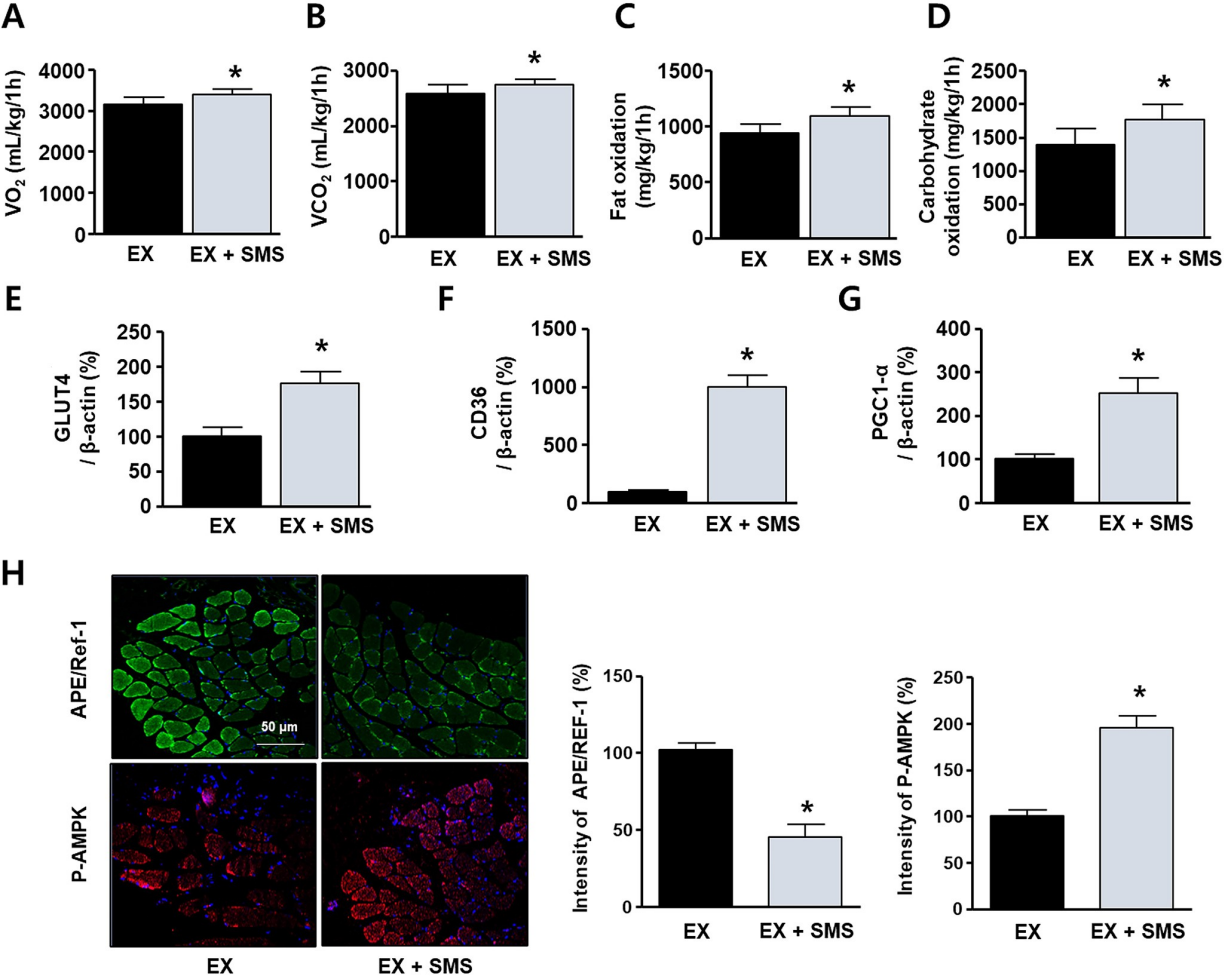
Regulation of energy metabolism by SMS during exercise. Mice were orally administered distilled water (EX) or 10 mL/kg of SMS (EX + SMS) 30 min prior to exercise and then exercised for 1 h. Changes in (A) O_2_ uptake, (B) CO_2_ production, and the sum of (C) lipid oxidation and (D) carbohydrate oxidation in the EX and EX + SMS groups for 1 h during exercise (n = 12). The mice were sacrificed post-exercise. mRNA expression levels in mice gastrocnemius muscles of GLUT4 (E), CD36 (F), and PGC1-α (G) normalized to that of β-actin. (H) Images of tissues sections (5 μm thick) incubated with anti-APE/Ref-1 (green) and anti-phospho-AMPK (red) antibodies, as well as DAPI (blue), and quantification of protein expression. All data are expressed as mean ± SD. *P < 0.05 compared to the EX group.

Gas respiratory analysis and immunohistochemistry were performed to evaluate energy metabolism during acute endurance exercise. Mice were pretreated with SMS 30 min prior to exercise and then exercised on a treadmill in a metabolic chamber for 1 h. Respiratory gas analysis was carried out to investigate the effects of SMS on the regulation of O_2_ uptake, CO_2_ production, carbohydrate oxidation, and fat oxidation during exercise. The SMS group exhibited significantly higher O_2_ uptake (Fig 3A; S3 Table), CO_2_ production (Fig 3B; S3 Table), and fat and carbohydrate oxidation (Fig 3C and 3D; S3 Table) than that of the EX group (*P* < 0.05). Analysis using qPCR indicated that gene expression of energy metabolic factors GLUT4, CD36, and PGC-1α significantly increased in skeletal muscles that were treated with SMS (Fig 3E–3G; S3 Table). Immunohistochemistry was performed to determine whether SMS reduced ROS generation in mice during acute exercise. A significant downregulation of APE/Ref-1 expression was detected in the gastrocnemius muscles of animals in the EX + SMS group (Fig 3H; S3 Table). The expression of AMPK-phosphorylated proteins was measured to confirm SMS-mediated activation of energy metabolism. SMS co-treatment showed a significantly elevated P-AMPK (192.8 ± 13.7%) level compared with that in the EX-treated group.

### Evaluation of changes in glucose/lipid metabolism in skeletal muscles during acute exercise

**Fig 4:**
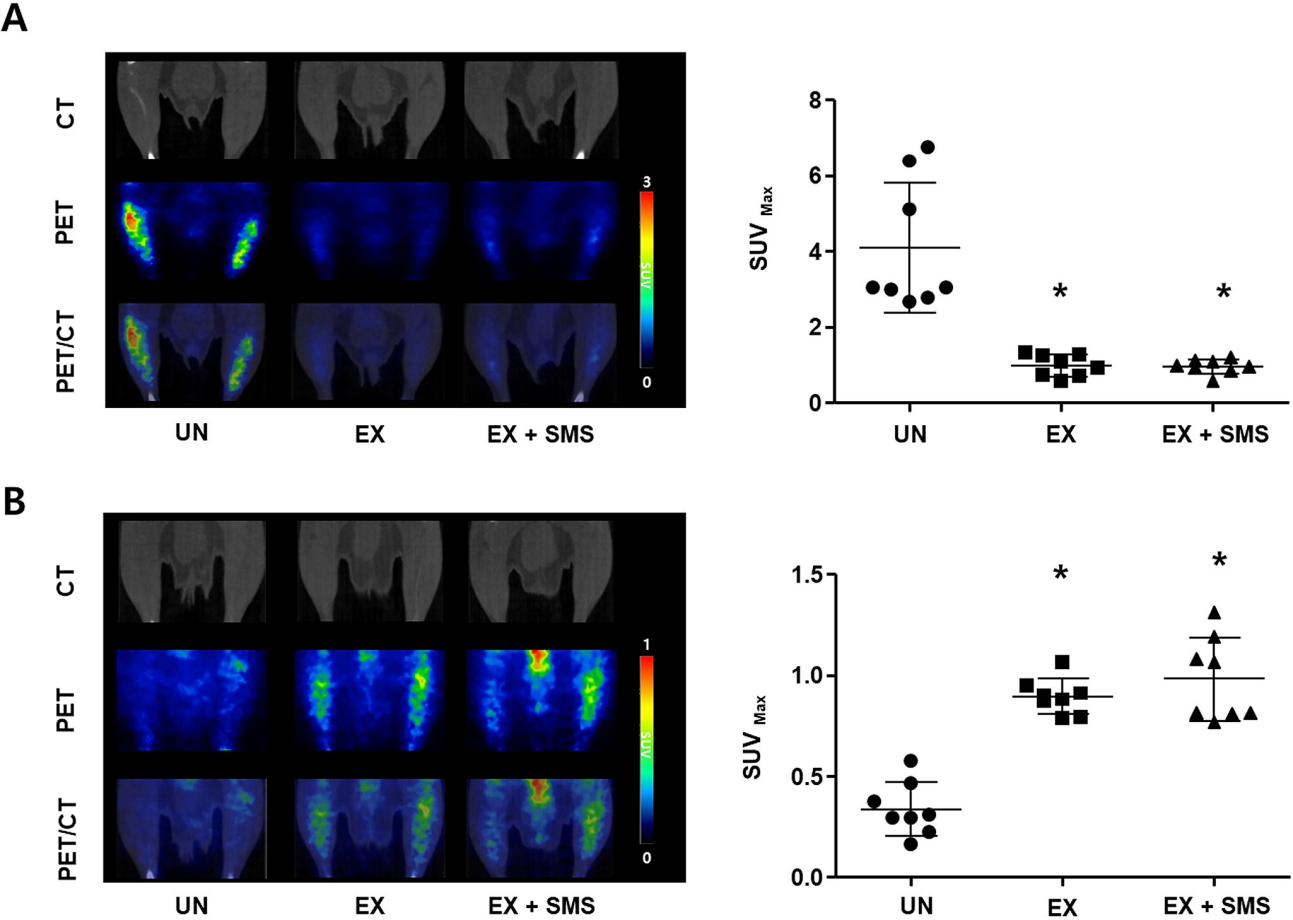
^18^F-FCH and ^18^F-FDG PET-CT uptake analyses in skeletal muscles. Mice were divided randomly into the following groups: untreated (UN), oral administration of water and exercise (EX), and oral administration of SMS (10 mL/kg) and exercise (EX + SMS), and subjected to 1 h of exercise. PET/CT images of (A) glucose metabolism and (B) lipid metabolism using ^18^F-FDG and ^18^F-FCH, respectively, and quantification of the SUV_max_ of the radiotracers in skeletal muscles. All data are expressed as mean ± SD. *P < 0.05 compared to the UN group.

Dedicated small-animal PET/CT imaging based on ^18^F-FCH and ^18^F-FDG uptake was performed to visualize and quantify lipid and glucose metabolism in skeletal muscles, respectively. The mean SUV_max_ value determined using ^18^F-FDG-PET/CT indicated that metabolism was markedly decreased in mice after endurance exercise on a treadmill (Fig 4A; S4 Table; 0.99 ± 0.29 for EX, 0.95 ± 0.19 for EX + SMS, and 4.09 ± 1.71 for UN). The ^18^F-FCH uptake value, used to access lipid metabolism in the skeletal muscles of acutely exercised mice, significantly increased from 0.34 ± 0.13 in the UN group to 0.89 ± 0.09 and 0.98 ± 0.21 in the EX and EX + SMS groups, respectively (Fig 4B; S4 Table). In contrast, SMS administration had no significant effect on the uptake of either compound.

## Discussion

Traditional oriental medicines (TOMs) have been widely used clinically in various Asian countries to treat numerous diseases [19]. Although several TOMs are used to treat fatigue and improve stamina, combination herbal medicines containing GS are rarely used owing to the lack of scientific evidence on their pharmacological effects and underlying molecular mechanisms. Therefore, this study aimed to investigate the mechanism(s) underlying the regulation of energy metabolism by prescription SMS. In modern society, the main causes of disease outbreaks are sedentary lifestyle, excessive food intake, and nutritional imbalances [20]. Controlling energy metabolism imbalances in the body and suppressing the occurrence of oxidative stress, which inevitably occurs during energy metabolism, are vital for disease control. In this study, we found that GS alone and SMS exhibited regulatory effects on oxidative stress and energy metabolism in L6 skeletal muscle cells. Our results imply that SMS inhibits oxidative stress by reducing ERK 1/2 and p38 phosphorylation. Furthermore, SMS significantly modulates energy metabolism by regulating lipid accumulation and gluconeogenesis. Although SMS affects physiological functions and other functions, its effects on energy metabolism have not been established to date. The results of this study provide a better understanding of in vivo respiratory metabolism in exercised mice and molecular biology studies in high fat-fed mice. Therefore, we suggest that SMS can be prescribed to enhance energy metabolism through its regulation of incomplete metabolism and oxidative stress.

Continuous muscle contraction due to physical activity can improve and prevent various metabolic diseases by increasing the energy demand of skeletal muscles [21]. Fats and carbohydrates are oxidized as a mixture and are the predominant fuels that affect various aspects of exercise, including intensity, type, training condition, and duration [22]. However, oxidative stress is inherently generated by excessive oxygen demand during prolonged high-intensity or acute untrained exercise. Some researchers have suggested that ongoing research on exercise supplements can effectively prevent ROS generation due to increasing energy expenditure [23]. Therefore, this study aimed to investigate energy metabolism at the molecular level in SMS-treated mice. In aerobic organisms, ROS are inevitably produced during respiratory processes as part of the physiological and metabolic processes [24]. Metabolic dysfunction, oxidative stress, and lipid peroxidation due to excessive exercise can lead to physical fatigue [25]. Oxidative stress is caused by an imbalance in ROS production relative to ROS depletion, which can lead to various adverse effects in the body [26]. In this study, SMS treatment significantly reduced the mitochondrial damage caused by exogenous H_2_O_2_ in L6 cells (Fig 2). Oxidative stress induced by ROS activates the MAPK signaling cascade, thereby leading to excessive ROS-mediated damage to the muscles [27]. SMS treatment significantly reduced H_2_O_2_-induced MAPK signaling (Fig 2), suggesting that SMS may exert protective effects against oxidative stress. Furthermore, we found that the molecular mechanism underlying the anti-fatigue effects of SMS is related to the regulation of energy metabolism and oxidative stress-induced expression of APE/Ref-1 and P-AMPKs. This implies that SMS not only participates in the attenuation of mitochondrial dysfunction, but it also promotes fat and carbohydrate metabolism. Therefore, we suggest that SMS can be used as a natural prescription medicine to suppress ROS production and oxidative stress caused by physical activity, promote energy metabolism, and provide anti-fatigue effects.

During strenuous exercise, gluconeogenesis is associated with non-carbohydrate carbon substances such as lactic acid and glycerol [28]. An earlier study found that four weeks of swimming combined with SMS consumption improved total exercise time (min) by approximately 10% (Effect size Cohen’s d; 1.81). In addition, the percentage of body fat significantly decreased compared to that in the control group (*P* < 0.001). However, this study was unable to establish the mechanism underlying endurance improvement by SMS during exercise, and further studies on fatigue-related substances, immune response markers, and blood lipids are needed to support these findings [29]. Therefore, we focused on the effects of SMS on energy metabolism during exercise by investigating the post-effects of SMS administration 30 min prior to exercise. After 1 h of exercising, O_2_ uptake and CO_2_ production were significantly higher in the SMS group than the UN group (*P* < 0.05). SMS intake before exercise appeared to boost energy metabolism by promoting carbohydrate and fat conversion (Fig 3). Buschiazzo *et al*. reported that in the fasting state, glucose metabolism in muscles rapidly decreases, resulting in increased ^18^F-FDG uptake [30]. Our results also indicated that SMS treatment prior to exercise significantly reduced in vitro and in vivo ^18^F-FDG uptake (Fig 4). Interestingly, there were no significant differences in ^18^F-FDG and ^18^F-FCH uptake after exercise upon SMS treatment. We hypothesized that glucose and lipid metabolism were depleted presumably due to rapid changes in energy metabolism during exercise (SMS treatment may not have caused any detectable changes in ^18^F-FDG or ^18^F-FCH uptake). We also suggest that owing to the increase in energy consumption during exercise post-SMS treatment, long-term SMS intake in combination with regular exercise may reduce body fat. Therefore, SMS may be a potential ergogenic aid to improve exercise capacity via an increase in energy consumption during exercise.

## Conclusions

In the present study, we demonstrated that the polyherbal medicine, SMS which contains GS, can exhibit multiple physiological regulatory activities in mice during exercise. SMS significantly attenuated P-p38 and ERK 1/2 expression, and inhibited the effect of mitochondrial dysfunction under ROS-mediated oxidative stress conditions on fat/carbohydrate metabolism in mice skeletal muscles. Our results suggest that SMS can exhibit radical scavenging activity against oxidative stress-related skeletal muscle damage and fat and carbohydrate energy metabolism during exercise. Therefore, SMS may be used as an exercise supplement or a prescription medicine to enhance energy metabolism and effectively inhibit ROS production during exercise.

## Supporting information

S1 Table Data set for effect of SMS on glucose and fat metabolism in L6 cells. (PDF)

S2 Table Data set for antioxidant effects of GS and SMS on H_2_O_2_-stimulated L6 cells. (PDF)

S3 Table Data set for regulation of energy metabolism by SMS during exercise. (PDF)

S4 Table Data set for ^18^F-FCH and ^18^F-FDG PET-CT uptake analyses in skeletal muscles. (PDF)

S1 Raw images (PDF)

S1 File Supplemental figures. (PDF)

S2 File (PDF)
