# Supplementary material for: Saengmaeksan, a traditional polyherbal formulation containing Panax ginseng, improves energy metabolism during exercise
Source: PLoS One. 2024 Jan 29;19(1):e0296487. doi: 10.1371/journal.pone.0296487 (PMC10824426; doi:10.1371/journal.pone.0296487)
Supplement: S1 Table — (PDF) [file pone.0296487.s001.pdf]

S1 Table.

Data set for effect of SMS on glucose and fat metabolism in L6 cells

Figure 1A.

	UN, %ID			
	10 min	30 min	60 min	120 min
	0.26	0.44	0.98	2.02
	0.28	0.45	1.03	1.78
	0.28	0.42	1.11	1.93
AVERAGE	0.27	0.44	1.04	1.91
STDEV	0.01	0.02	0.07	0.12

	GS, %ID			
	10 min	30 min	60 min	120 min
	0.28	0.49	1.00	1.55
	0.29	0.43	1.07	1.83
	0.28	0.56	1.02	1.63
AVERAGE	0.28	0.49	1.03	1.67
STDEV	0.01	0.07	0.04	0.14

	LM, %ID			
	10 min	30 min	60 min	120 min
	0.20	0.38	0.82	1.30
	0.21	0.32	0.83	1.50
	0.20	0.34	0.82	1.42
AVERAGE	0.20	0.35	0.82	1.41
STDEV	0.01	0.03	0.01	0.10

	SC , %ID			
	10 min	30 min	60 min	120 min
	0.26	0.37	0.97	1.49
	0.25	0.35	0.91	1.50
	0.28	0.32	1.06	1.51
AVERAGE	0.26	0.35	0.98	1.50
STDEV	0.02	0.03	0.08	0.01

	GS+LM, %ID			
	10 min	30 min	60 min	120 min
	0.26	0.36	0.74	1.34
	0.25	0.37	0.78	1.45
	0.25	0.46	0.87	1.44
AVERAGE	0.25	0.40	0.80	1.41
STDEV	0.01	0.06	0.07	0.06

	GS+SC, %ID			
	10 min	30 min	60 min	120 min
	0.25	0.47	0.75	1.35
	0.27	0.42	0.81	1.67
	0.28	0.46	0.78	1.69
AVERAGE	0.27	0.45	0.78	1.57
STDEV	0.02	0.03	0.03	0.19

	LM+SC, %ID			
	10 min	30 min	60 min	120 min
	0.26	0.40	0.91	1.61
	0.28	0.38	0.95	1.58
	0.28	0.44	0.95	1.60
AVERAGE	0.27	0.41	0.94	1.60
STDEV	0.01	0.03	0.02	0.02

	GS+LM+SC, %ID			
	10 min	30 min	60 min	120 min
	0.30	0.36	0.82	0.67
	0.28	0.39	0.79	0.69
	0.28	0.33	0.78	0.69
AVERAGE	0.29	0.36	0.80	0.69
STDEV	0.01	0.03	0.02	0.01

Figure 1B.

	UN	GS	SMS
	0.35	0.32	0.12
	0.26	0.13	0.14
	0.36	0.09	0.04
	0.20	0.26	0.19
	0.21	0.10	0.06
	0.13	0.15	0.05
	0.22	0.10	0.18
AVERAGE	0.25	0.16	0.11
STDEV	0.08	0.09	0.06

Figure 1C.

	UN	GS	SMS
	98.3	153.3	177.0
	100.4	160.8	150.3
	101.4	182.9	180.9
AVERAGE	100.0	165.7	169.4
STDEV	1.6	15.4	16.6

Figure 1D.

	CPT1		
	UN	GS	SMS
	113.3	156.9	577.6
	101.4	174.1	446.9
	87.1	184.0	389.1
AVERAGE	100.6	171.7	471.2
STDEV	13.1	13.7	96.6

	CD36		
	UN	GS	SMS
	78.3	781.7	803.7
	143.1	671.2	755.1
	89.3	639.4	675.8
AVERAGE	103.5	697.4	744.9
STDEV	34.7	74.7	64.5

	Glut4		
	UN	GS	SMS
	83.9	61.8	58.9
	122.8	84.5	32.0
	97.0	91.8	60.6
AVERAGE	101.3	79.4	50.5
STDEV	19.8	15.6	16.0

	MCT1		
	UN	GS	SMS
	92.0	124.0	115.7
	120.6	100.0	100.7
	90.1	111.7	99.3
AVERAGE	100.9	111.9	105.2
STDEV	17.1	12.0	9.1
