# Supplementary material for: Saengmaeksan, a traditional polyherbal formulation containing Panax ginseng, improves energy metabolism during exercise
Source: PLoS One. 2024 Jan 29;19(1):e0296487. doi: 10.1371/journal.pone.0296487 (PMC10824426; doi:10.1371/journal.pone.0296487)
Supplement: S2 Table — (PDF) [file pone.0296487.s002.pdf]

S2 Table.

Data set for antioxidant effects of GS and SMS on H2O2-stimulated L6 cells

Figure 2A.

	UN		
	UN	GS	SMS
	103.4	103.1	105.3
	98.5	99.1	109.0
	110.1	90.2	98.2
AVERAGE	104.0	97.4	104.1
STDVE	5.9	6.6	5.5

	H_2O_2		
	UN	GS	SMS
	70.3	89.0	87.0
	72.9	93.0	81.0
	73.9	83.0	79.0
AVERAGE	72.4	88.3	82.3
STDVE	1.9	5.0	4.2

Figure 2B.

	UN	H_2O_2	H_2O_2+GS	H_2O_2+SMS
	6.1	3.0	5.2	5.4
	7.8	2.8	5.4	6.0
	7.9	1.8	5.6	5.3
AVERAGE	7.3	2.5	5.4	5.5
STDEV	1.0	0.6	0.2	0.4

Figure 2C.

	UN	H_2O_2	H_2O_2+GS	H_2O_2+SMS
	98.4	208.0	126.3	122.3
	103.1	199.8	128.5	149.9
	98.5	193.2	136.4	133.4
AVERAGE	100.0	200.4	130.4	135.2
STDEV	2.7	7.4	5.3	13.9

Figure 2D.

	UN	H_2O_2	H_2O_2+GS	H_2O_2+SMS
	99.2	167.6	112.0	151.3
	103.6	176.4	123.2	127.2
	97.3	178.5	124.8	131.7
AVERAGE	100.0	174.2	120.0	136.7
STDEV	3.2	5.8	7.0	12.8

Figure 2E.

	p-ERK1/2				
	UN	H_2O_2	H_2O_2+SMS	H_2O_2+PD	H_2O_2+SB
	117.2	200.0	154.2	54.2	54.1
	87.7	180.1	166.8	52.9	60.2
	95.1	192.9	145.1	50.1	52.1
AVERAGE	100.0	191.0	155.4	52.4	55.5
STDEV	15.4	10.1	10.9	2.1	4.2

	p-p38				
	UN	H_2O_2	H_2O_2+SMS	H_2O_2+PD	H_2O_2+SB
	104.5	201.0	93.7	152.7	68.2
	94.6	180.3	116.1	176.0	60.8
	100.8	209.7	95.6	141.9	40.4
AVERAGE	100.0	197.0	101.8	156.9	56.5
STDEV	5.0	15.1	12.4	17.4	14.4

	APE/Ref-1				
	UN	H_2O_2	H_2O_2+SMS	H_2O_2+PD	H_2O_2+SB
	92.1	206.6	93.9	146.2	181.3
	113.2	192.0	117.1	130.8	161.2
	94.7	210.3	102.0	130.3	143.1
AVERAGE	100.0	202.9	104.3	135.7	161.9
STDEV	11.5	9.7	11.8	9.1	19.1
