# Supplementary material for: Saengmaeksan, a traditional polyherbal formulation containing Panax ginseng, improves energy metabolism during exercise
Source: PLoS One. 2024 Jan 29;19(1):e0296487. doi: 10.1371/journal.pone.0296487 (PMC10824426; doi:10.1371/journal.pone.0296487)
Supplement: S3 Table — (PDF) [file pone.0296487.s003.pdf]

S3 Table.

Data set for regulation of energy metabolism by SMS during exercise

Figure 3A.

	VO$_2$	
	EX	EX+SMS
	3,105.3	3,354.2
	3,109.5	3,314.9
	3,130.5	3,391.1
	3,191.4	3,623.8
	2,899.9	3,326.1
	3,430.1	3,314.8
AVERAGE	3,144.5	3,387.5
STEDV	171.2	119.4

Figure 3B.

	VCO$_2$	
	EX	EX+SMS
	2,705.9	2,769.7
	2,438.6	2,668.1
	2,563.5	2,649.5
	2,701.9	2,890.7
	2,342.9	2,746.9
	2,774.3	2,798.3
AVERAGE	2,587.9	2,753.9
STEDV	170.0	88.6

Figure 3C.

	Fat	
	EX	EX+SMS
	997.4	1,075.2
	1,005.7	1,080.3
	944.1	1,002.0
	813.5	1,225.3
	891.0	1,091.4
	995.0	1,099.0
AVERAGE	941.1	1,095.5
STEDV	76.2	72.5

Figure 3D.

	Carbohydrate	
	EX	EX+SMS
	1,182.9	1,606.8
	1,181.8	1,539.4
	1,657.7	1,853.7
	1,192.8	1,563.5
	1,461.6	1,864.4
	1,654.5	2,136.6
AVERAGE	1,388.5	1,760.7
STEDV	233.2	233.3

Figure 3E.

	GLUT4	
	EX	EX+SMS
	113.8	191.4
	97.7	178.6
	89.9	158.7
AVERAGE	100.5	176.2
STEDV	12.2	16.5

Figure 3F.

	CD36	
	EX	EX+SMS
	113.8	1,024.4
	95.7	1,082.8
	91.8	891.8
AVERAGE	100.4	999.6
STEDV	11.7	97.9

Figure 3G.

	PGC1-α	
	EX	EX+SMS
	113.0	226.1
	97.0	292.1
	91.2	234.0
AVERAGE	100.4	250.7
STEDV	11.3	36.1

Figure 3H.

	APE/RPF-1	
	EX	EX+SMS
	99.3	39.5
	96.2	54.1
	104.5	39.8
AVERAGE	100.0	44.5
STEDV	4.2	8.4

	P-AMPK	
	EX	EX+SMS
	99.4	208.3
	92.2	182.3
	108.4	187.6
AVERAGE	100.0	192.8
STEDV	8.1	13.7
