# Supplementary material for: Saengmaeksan, a traditional polyherbal formulation containing Panax ginseng, improves energy metabolism during exercise
Source: PLoS One. 2024 Jan 29;19(1):e0296487. doi: 10.1371/journal.pone.0296487 (PMC10824426; doi:10.1371/journal.pone.0296487)
Supplement: S4 Table — (PDF) [file pone.0296487.s004.pdf]

S4 Table.

Data set for ^{18}F-FCH and ^{18}F-FDG PET-CT uptake analyses in skeletal muscles

Figure 4A.

	[^{18}F]FDG, %ID/g		
	CTL	EX	EX+SMS
	3.05	1.26	1.11
	3.04	1.28	0.56
	5.10	1.08	0.97
	6.74	0.91	0.96
	2.66	0.72	0.93
	2.77	1.32	1.19
	2.98	0.58	1.07
	6.38	0.74	0.84
AVERAGE	4.09	0.99	0.95
STDEV	1.71	0.29	0.19

Figure 4B.

	[^{18}F]FCH, %ID/g		
	CTL	EX	EX+SMS
	0.22	0.87	1.06
	0.31	1.06	0.81
	0.29	0.91	1.08
	0.16	0.79	0.81
	0.58	0.79	0.77
	0.46	0.95	0.81
	0.29	0.90	1.31
	0.38	0.88	1.19
AVERAGE	0.34	0.89	0.98
STDEV	0.13	0.09	0.21
