# Supplementary figures and images for: Saengmaeksan, a traditional polyherbal formulation containing Panax ginseng, improves energy metabolism during exercise
Source: PLoS One. 2024 Jan 29;19(1):e0296487. doi: 10.1371/journal.pone.0296487 (PMC10824426; doi:10.1371/journal.pone.0296487)

# P-ERK 1/2

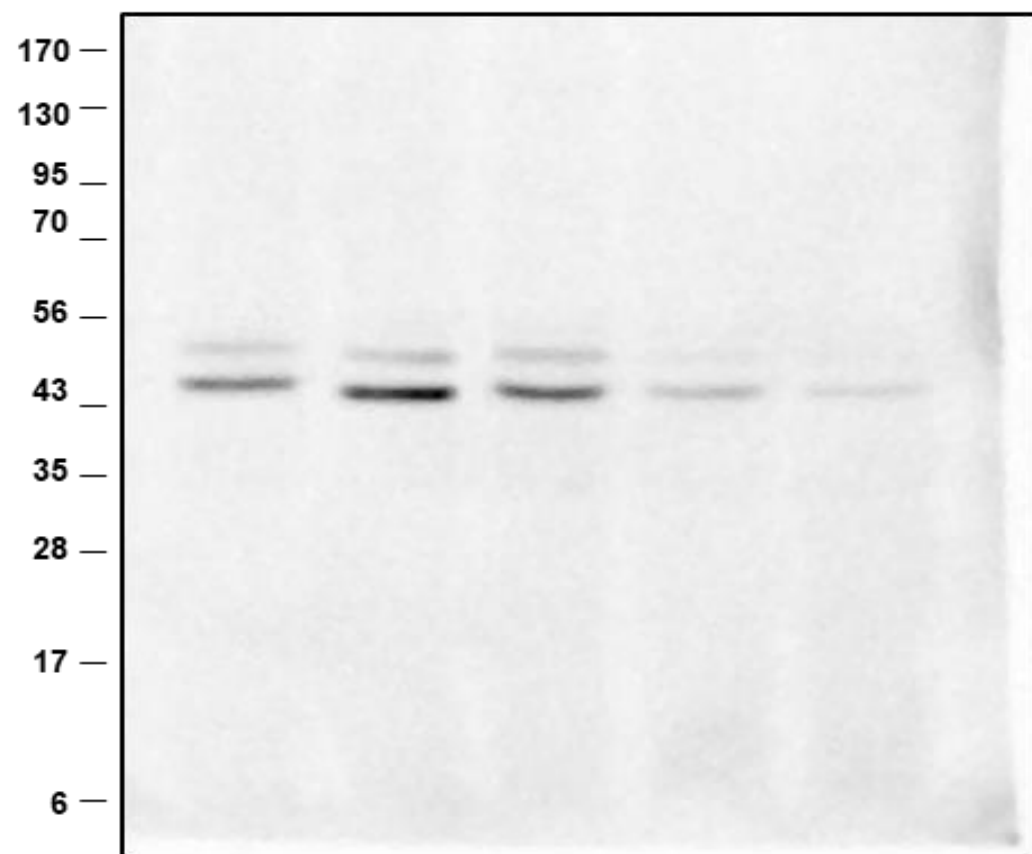

# T-ERK 1/2

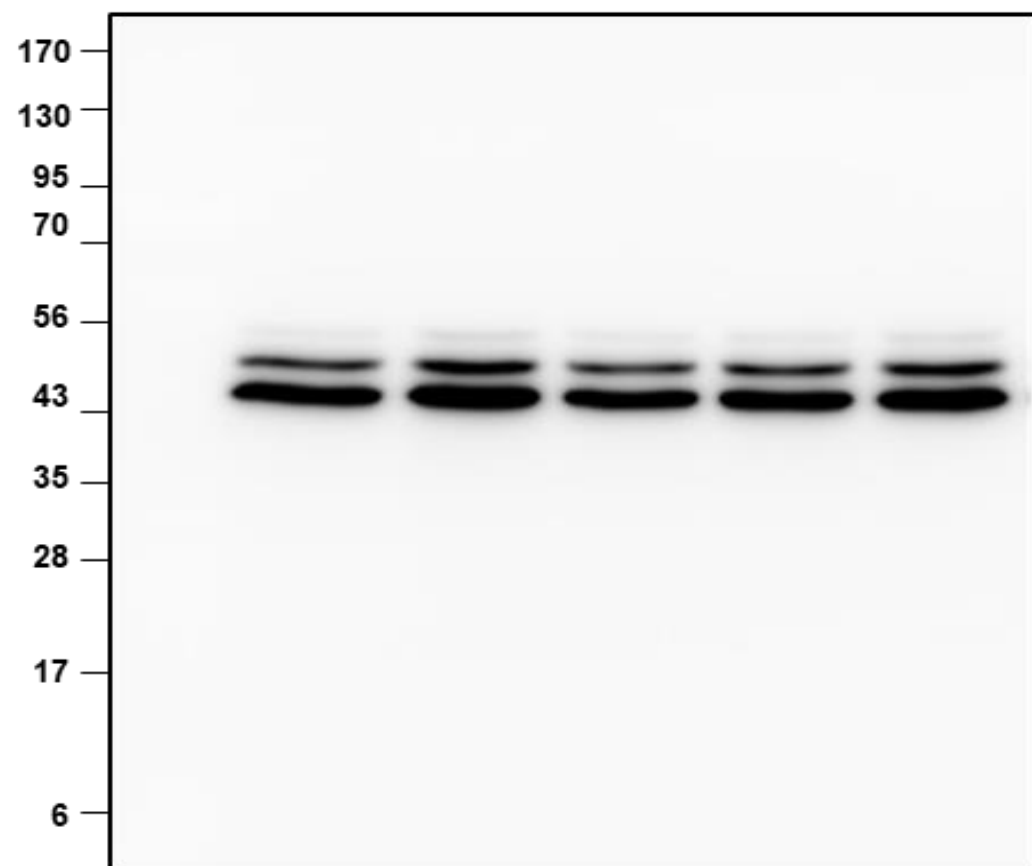

**P-p38**

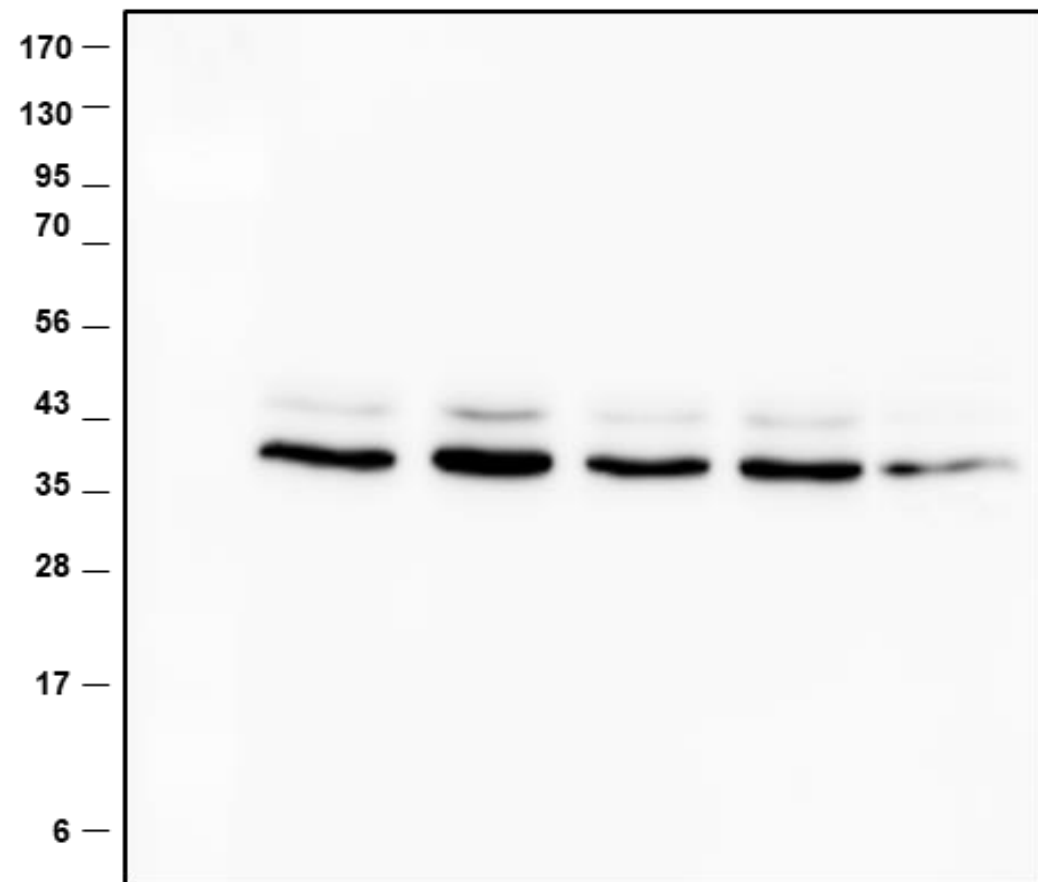

T-p38

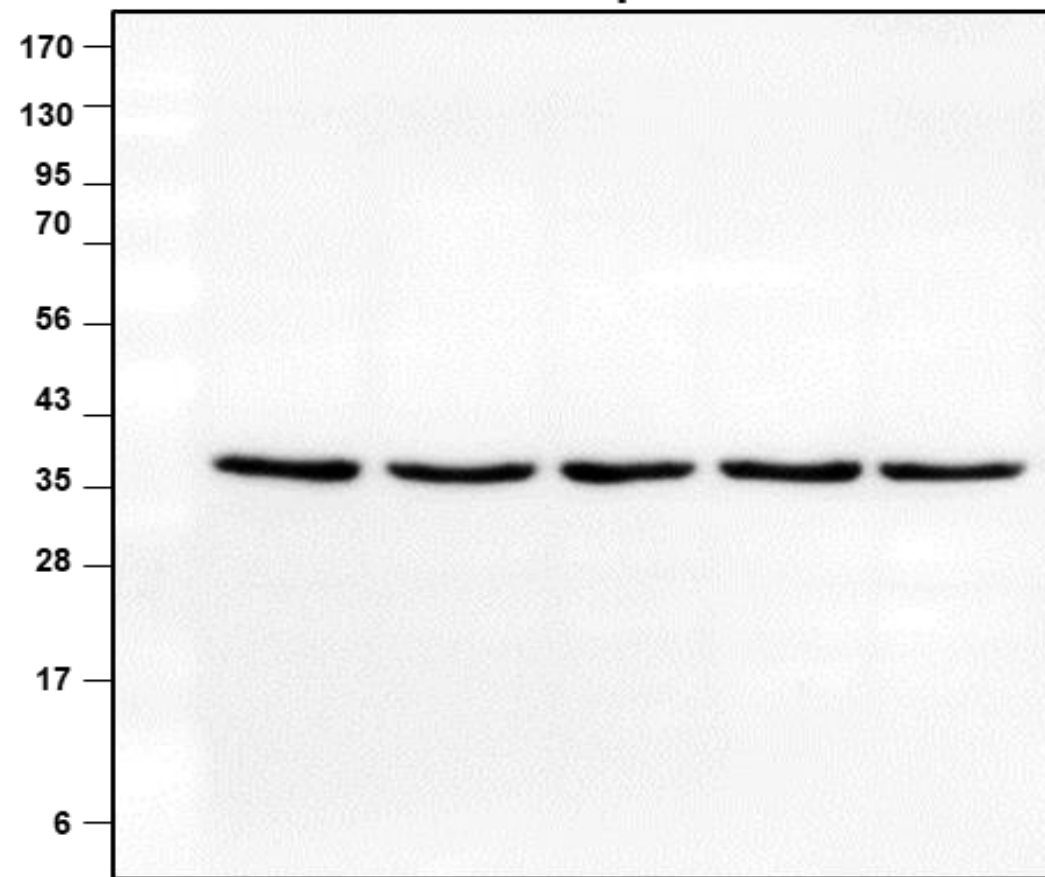

# APE/Ref-1

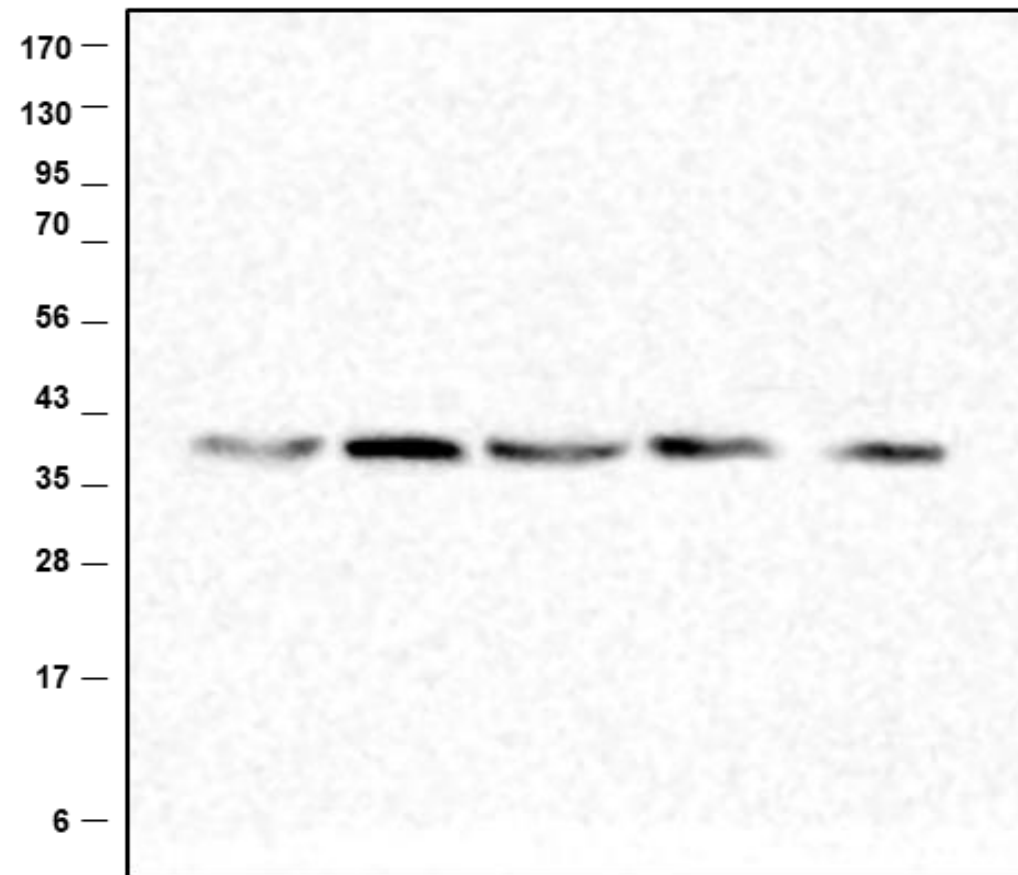

$\beta$ -actin

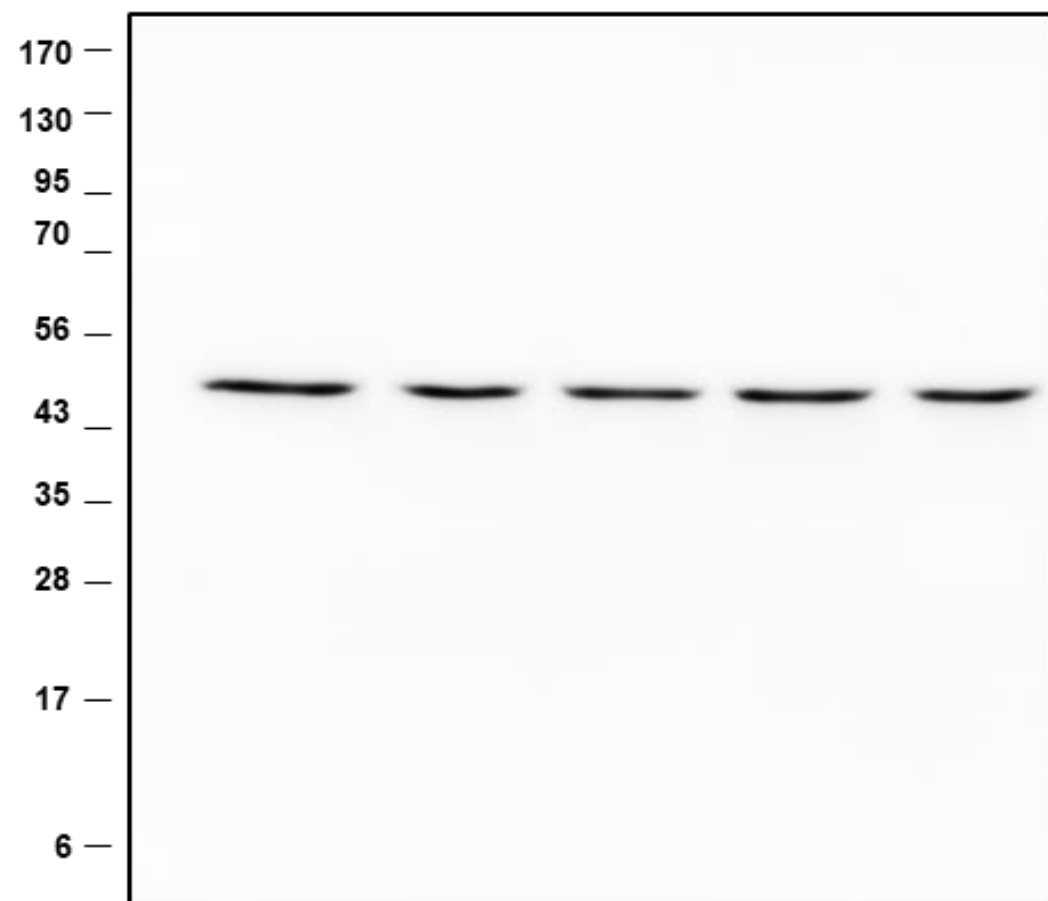

Supplement: S1 Raw images — (PDF) [file pone.0296487.s005.pdf]
