# Supplementary material for: Saengmaeksan, a traditional polyherbal formulation containing Panax ginseng, improves energy metabolism during exercise
Source: PLoS One. 2024 Jan 29;19(1):e0296487. doi: 10.1371/journal.pone.0296487 (PMC10824426; doi:10.1371/journal.pone.0296487)
Supplement: S1 File — (PDF) [file pone.0296487.s006.pdf]

Supplementary Information

S1 File.

In vitro glucose uptake analysis in myoblast C2C12 cells using ^{18}F-FDG.

C2C12 cells (2 × 10^5 cells/well) were seeded in 6-well culture plates and treated with 0.125 mg/mL GS or 0.5 mg SMS (mixture of GS, LM and SC in the ratio of 1:2:1) in DMEM medium. After 24 h, the cells were washed using phosphate buffered saline (PBS), treated with 370 kilobecquerel (kBq) of ^{18}F-FDG/mL serum free medium and then incubated for 120 min at 37 °C in 5% CO_2 atmosphere. The medium was removed and the cells were washed with PBS. The adherent cells were harvested using Trypsin and their radioactivity was determined using a 2470 Wizard2 gamma counter (PerkinElmer, MA, USA).

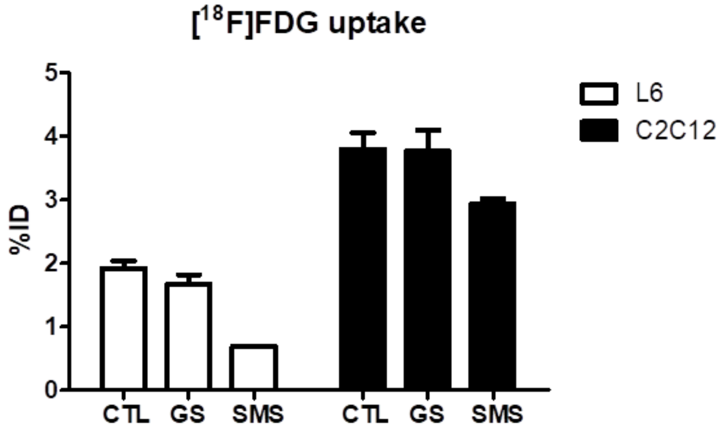

S1 Figure. In vitro glucose uptake analysis in myoblast C2C12 cells using ^{18}F-FDG.

Data set for S1 Figure.

	L6, %ID		
	CTL	GS	SMS
	2.02	1.55	0.67
	1.78	1.83	0.69
	1.93	1.63	0.69
AVERAGE	1.91	1.67	0.68
STDEV	0.12	0.14	0.01

	C2C12, %ID		
	CTL	GS	SMS
	4.20	4.29	2.90
	3.99	3.78	3.02
	3.18	3.24	2.88
AVERAGE	3.79	3.77	2.94
STDEV	0.54	0.53	0.07

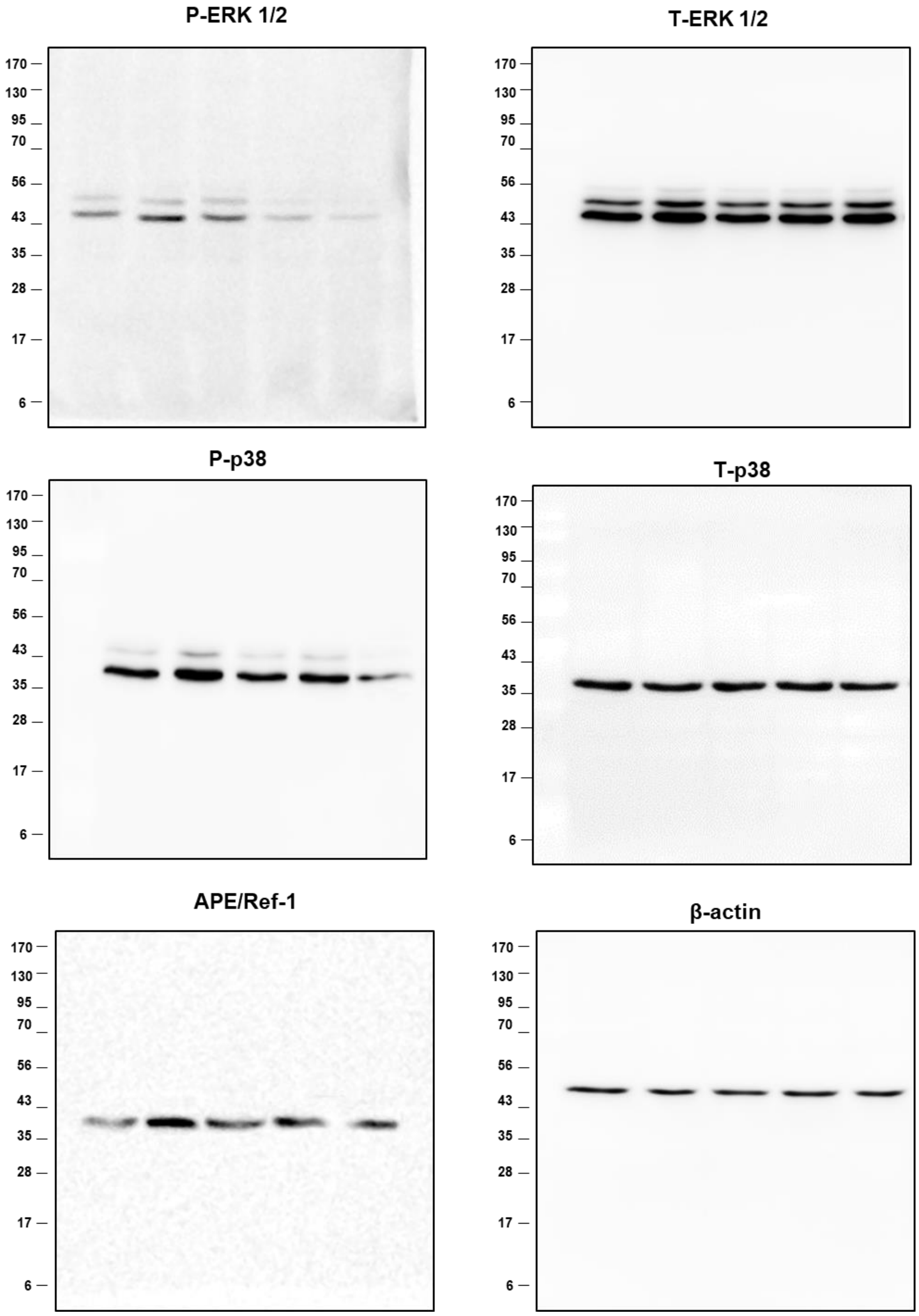

S2 Figure. The full blot results of Western blot analysis.
