# Supplementary material for: Saengmaeksan, a traditional polyherbal formulation containing Panax ginseng, improves energy metabolism during exercise
Source: PLoS One. 2024 Jan 29;19(1):e0296487. doi: 10.1371/journal.pone.0296487 (PMC10824426; doi:10.1371/journal.pone.0296487)
Supplement: S2 File — (PDF) [file pone.0296487.s007.pdf]

Figure 1A

	UN, %ID			
	10 min	30 min	60 min	120 min
	0.26	0.44	0.98	2.02
	0.28	0.45	1.03	1.78
	0.28	0.42	1.11	1.93
AVERAGE	0.27	0.44	1.04	1.91
STDEV	0.01	0.02	0.07	0.12

	GS, %ID			
	10 min	30 min	60 min	120 min
	0.28	0.49	1.00	1.55
	0.29	0.43	1.07	1.83
	0.28	0.56	1.02	1.63
AVERAGE	0.28	0.49	1.03	1.67
STDEV	0.01	0.07	0.04	0.14

	LM, %ID			
	10 min	30 min	60 min	120 min
	0.20	0.38	0.82	1.30
	0.21	0.32	0.83	1.50
	0.20	0.34	0.82	1.42
AVERAGE	0.20	0.35	0.82	1.41
STDEV	0.01	0.03	0.01	0.10

	SC , %ID			
	10 min	30 min	60 min	120 min
	0.26	0.37	0.97	1.49
	0.25	0.35	0.91	1.50
	0.28	0.32	1.06	1.51
AVERAGE	0.26	0.35	0.98	1.50
STDEV	0.02	0.03	0.08	0.01

	GS+LM, %ID			
	10 min	30 min	60 min	120 min
	0.26	0.36	0.74	1.34
	0.25	0.37	0.78	1.45
	0.25	0.46	0.87	1.44
AVERAGE	0.25	0.40	0.80	1.41
STDEV	0.01	0.06	0.07	0.06

	GS+SC, %ID			
	10 min	30 min	60 min	120 min
	0.25	0.47	0.75	1.35
	0.27	0.42	0.81	1.67
	0.28	0.46	0.78	1.69
AVERAGE	0.27	0.45	0.78	1.57
STDEV	0.02	0.03	0.03	0.19

	LM+SC, %ID			
	10 min	30 min	60 min	120 min
	0.26	0.40	0.91	1.61
	0.28	0.38	0.95	1.58
	0.28	0.44	0.95	1.60
AVERAGE	0.27	0.41	0.94	1.60
STDEV	0.01	0.03	0.02	0.02

	GS+LM+SC, %ID			
	10 min	30 min	60 min	120 min
	0.30	0.36	0.82	0.67
	0.28	0.39	0.79	0.69
	0.28	0.33	0.78	0.69
AVERAGE	0.29	0.36	0.80	0.69
STDEV	0.01	0.03	0.02	0.01

Figure 1B

	UN	GS	SMS
	0.35	0.32	0.12
	0.26	0.13	0.14
	0.36	0.09	0.04
	0.20	0.26	0.19
	0.21	0.10	0.06
	0.13	0.15	0.05
	0.22	0.10	0.18
AVERAGE	0.25	0.16	0.11
STDEV	0.08	0.09	0.06

Figure 1C

	UN	GS	SMS
	98.3	153.3	177.0
	100.4	160.8	150.3
	101.4	182.9	180.9
AVERAGE	100.0	165.7	169.4
STDEV	1.6	15.4	16.6

Figure 1D

	CPT1		
	UN	GS	SMS
	113.3	156.9	577.6
	101.4	174.1	446.9
	87.1	184.0	389.1
AVERAGE	100.6	171.7	471.2
STDEV	13.1	13.7	96.6

	CD36		
	UN	GS	SMS
	78.3	781.7	803.7
	143.1	671.2	755.1
	89.3	639.4	675.8
AVERAGE	103.5	697.4	744.9
STDEV	34.7	74.7	64.5

	Glut4		
	UN	GS	SMS
	83.9	61.8	58.9
	122.8	84.5	32.0
	97.0	91.8	60.6
AVERAGE	101.3	79.4	50.5
STDEV	19.8	15.6	16.0

	MCT1		
	UN	GS	SMS
	92.0	124.0	115.7
	120.6	100.0	100.7
	90.1	111.7	99.3
AVERAGE	100.9	111.9	105.2
STDEV	17.1	12.0	9.1

Figure 2A

	UN			H_2O_2		
	UN	GS	SMS	UN	GS	SMS
	103.4	103.1	105.3	70.3	89.0	87.0
	98.5	99.1	109.0	72.9	93.0	81.0
	110.1	90.2	98.2	73.9	83.0	79.0
AVERAGE	104.0	97.4	104.1	72.4	88.3	82.3
STDVE	5.9	6.6	5.5	1.9	5.0	4.2

Figure 2B

	UN	H_2O_2	H_2O_2+GS	H_2O_2+SMS
	6.1	3.0	5.2	5.4
	7.8	2.8	5.4	6.0
	7.9	1.8	5.6	5.3
AVERAGE	7.3	2.5	5.4	5.5
STDEV	1.0	0.6	0.2	0.4

Figure 2C

	UN	H_2O_2	H_2O_2+GS	H_2O_2+SMS
	98.4	208.0	126.3	122.3
	103.1	199.8	128.5	149.9
	98.5	193.2	136.4	133.4
AVERAGE	100.0	200.4	130.4	135.2
STDEV	2.7	7.4	5.3	13.9

Figure 2D

	UN	H_2O_2	H_2O_2+GS	H_2O_2+SMS
	99.2	167.6	112.0	151.3
	103.6	176.4	123.2	127.2
	97.3	178.5	124.8	131.7
AVERAGE	100.0	174.2	120.0	136.7
STDEV	3.2	5.8	7.0	12.8

Figure 2E

	p-ERK1/2				
	UN	H_2O_2	H_2O_2+SMS	H_2O_2+PD	H_2O_2+SB
	117.2	200.0	154.2	54.2	54.1
	87.7	180.1	166.8	52.9	60.2
	95.1	192.9	145.1	50.1	52.1
AVERAGE	100.0	191.0	155.4	52.4	55.5
STDEV	15.4	10.1	10.9	2.1	4.2

	p-p38				
	UN	H_2O_2	H_2O_2+SMS	H_2O_2+PD	H_2O_2+SB
	104.5	201.0	93.7	152.7	68.2
	94.6	180.3	116.1	176.0	60.8
	100.8	209.7	95.6	141.9	40.4
AVERAGE	100.0	197.0	101.8	156.9	56.5
STDEV	5.0	15.1	12.4	17.4	14.4

	APE/Ref-1				
	UN	H_2O_2	H_2O_2+SMS	H_2O_2+PD	H_2O_2+SB
	92.1	206.6	93.9	146.2	181.3
	113.2	192.0	117.1	130.8	161.2
	94.7	210.3	102.0	130.3	143.1
AVERAGE	100.0	202.9	104.3	135.7	161.9
STDEV	11.5	9.7	11.8	9.1	19.1

Figure 3A

	VO_2	
	EX	EX+SMS
	3,105.3	3,354.2
	3,109.5	3,314.9
	3,130.5	3,391.1
	3,191.4	3,623.8
	2,899.9	3,326.1
	3,430.1	3,314.8
AVERAGE	3,144.5	3,387.5
STEDV	171.2	119.4

Figure 3B

	VCO_2	
	EX	EX+SMS
	2,705.9	2,769.7
	2,438.6	2,668.1
	2,563.5	2,649.5
	2,701.9	2,890.7
	2,342.9	2,746.9
	2,774.3	2,798.3
AVERAGE	2,587.9	2,753.9
STEDV	170.0	88.6

Figure 3C

	Fat	
	EX	EX+SMS
	997.4	1,075.2
	1,005.7	1,080.3
	944.1	1,002.0
	813.5	1,225.3
	891.0	1,091.4
	995.0	1,099.0
AVERAGE	941.1	1,095.5
STEDV	76.2	72.5

Figure 3D

	Carbohydrate	
	EX	EX+SMS
	1,182.9	1,606.8
	1,181.8	1,539.4
	1,657.7	1,853.7
	1,192.8	1,563.5
	1,461.6	1,864.4
	1,654.5	2,136.6
AVERAGE	1,388.5	1,760.7
STEDV	233.2	233.3

Figure 3E

	GLUT4	
	EX	EX+SMS
	113.8	191.4
	97.7	178.6
	89.9	158.7
AVERAGE	100.5	176.2
STEDV	12.2	16.5

Figure 3F

	CD36	
	EX	EX+SMS
	113.8	1,024.4
	95.7	1,082.8
	91.8	891.8
AVERAGE	100.4	999.6
STEDV	11.7	97.9

Figure 3G

	PGC1-α	
	EX	EX+SMS
	113.0	226.1
	97.0	292.1
	91.2	234.0
AVERAGE	100.4	250.7
STEDV	11.3	36.1

Figure 3H

	APE/RPF-1	
	EX	EX+SMS
	99.3	39.5
	96.2	54.1
	104.5	39.8
AVERAGE	100.0	44.5
STEDV	4.2	8.4

	P-AMPK	
	EX	EX+SMS
	99.4	208.3
	92.2	182.3
	108.4	187.6
AVERAGE	100.0	192.8
STEDV	8.1	13.7

Figure 4A

	$[^{18}F]$FDG, %ID/g		
	CTL	EX	EX+SMS
	3.05	1.26	1.11
	3.04	1.28	0.56
	5.10	1.08	0.97
	6.74	0.91	0.96
	2.66	0.72	0.93
	2.77	1.32	1.19
	2.98	0.58	1.07
	6.38	0.74	0.84
AVERAGE	4.09	0.99	0.95
STDEV	1.71	0.29	0.19

Figure 4B

	$[^{18}F]$FCH, %ID/g		
	CTL	EX	EX+SMS
	0.22	0.87	1.06
	0.31	1.06	0.81
	0.29	0.91	1.08
	0.16	0.79	0.81
	0.58	0.79	0.77
	0.46	0.95	0.81
	0.29	0.90	1.31
	0.38	0.88	1.19
AVERAGE	0.34	0.89	0.98
STDEV	0.13	0.09	0.21

Figure S1

	L6, %ID		
	CTL	GS	SMS
	2.02	1.55	0.67
	1.78	1.83	0.69
	1.93	1.63	0.69
AVERAGE	1.91	1.67	0.68
STDEV	0.12	0.14	0.01

	C2C12, %ID		
	CTL	GS	SMS
	4.20	4.29	2.90
	3.99	3.78	3.02
	3.18	3.24	2.88
AVERAGE	3.79	3.77	2.94
STDEV	0.54	0.53	0.07
